# Original Communications

**Published:** 1844-10-01

**Authors:** 


### ORIGINAL COMMUNICATIONS.

### ON THE CAUSE OF ELASTICITY AND VARIOUS EFFECTS OF TENSION IN THE HUMAN FRAME. By *T. Wilkinson King*, F.R.C.S.E.

*Former Views of the Writer—Humoral Interferences—Dr. Todd and Mr. Bowman's Views of Tissue—Different Tunics of Bones, of Nerves, of Vessels, of Organs, of Ducts—Comparison of Cava and Saphena—Theory of Varix—Fasciæ—Blood-tensions.*

A PRETTY train of just thoughts even in philosophical matters may be far enough away from what is useful in practical life. To some minds, perhaps, the sense of beauty and utility are scarcely separable, and yet again there are very few who do not at once yield to the simple well-expressed hypothesis which is consonant with known facts and not opposed to established opinions. These ideas apply in a little measure to the subjoined view of tension and the cause of elasticity. The explanation may seem pretty, to some perhaps not useful; and it may appear just to many: yet there is room to fear that, after being admitted, it will be found to offend against doctrines long received. To these I shall not again refer; nor shall I refer to final causes. The object I have now in hand is chiefly to strengthen and to illustrate some already stated opinions by considering the mode of extension which induces elasticity.

I have elsewhere endeavoured to shew what tension has to do with the growth, excavation, repair and wasting of bone,* and with the evolution of some other tissues. To tension I have referred the new ligament that re-unites divided muscles, tendons and ligaments, as well as bones occasionally.† By tension I have endeavoured to explain the order of the initiative ossifications in the skeleton, and that of the consolidation of the various epiphyses of bones.§ To a certain degree and mode of extension it is that I suppose the elasticity of tissues to be due. The enquiry itself I am very far from thinking devoid of practical interest. It rests a claim on this; and the opinion, when established will, I think, conduce strongly to safe and more defined principles of treatment.

The matter which nourishes the soft tissues, decides no doubt, and in some parts more markedly than others, their measure of elasticity. Thus, the most healthy pleuritic bridle seems like pure reticular membrane, but various less natural adhesions put on tendinous or almost unorganized characters. So deteriorated blood makes ligaments ossify, but those first which are most strained. Such with the best nutrition would only grow strong; with abundant defective nourishment, and much use, they do but increase morbidly.

Ligaments, tendon, reticular tissue and skin, unite to secure the knee-joint. Deprive the solid cords of all tension and they disappear. Extend and nourish a band of elastic membrane or of skin, and the energy being sufficient it becomes

---

* See "Fracture," Enyclop. of Surg. and "Isolated New Bone," Prov. Med. Journal, Aug. 12, 1843.

† Ibid. and "Repair of Patella Broken Transversely," Guy's Hosp. Reports, No. 13.

§ London Med. Gaz. April 19, 1844. To the decline of tension I have imputed occasional isolations of parts of bone and the same likewise to excessive tensions.

tendon. Give it but a certain great or free activity and its mass and elasticity are increased.

Suppose a radius or a lever with equal parallel cords stretched to different points of its length; or, suppose cords of equal strength, but of different lengths, to be acted upon by one and the same force, and it seems almost self-evident that the same powers and qualities of organization (nutrition or repair) must operate differently on all of these. Thus it is that we find reticular tissue, skin, &c. varying all over the body.

I avail myself freely of the ingenious and valuable labours of Dr. Todd and Mr. Bowman, as it is almost necessary here to set forth the best knowledge we possess of the anatomy of the tissues in question.—(*See* "The Physiological Anatomy and Physiology of Man," Part I. p. 69, *et seq.*)

Ligamentous or fibrous tissues are two—the "white" and the "yellow." The first "it would appear incorrect to describe as a bundle of threads. It is rather a mass, with longitudinal parallel streaks (many of which are creasings) and which has a tendency to slit up almost ad infinitum." Acid obliterates "all" appearance of fibrillæ in it.

The yellow fibrous tissue consists "of fibres, round in some, flattened in other specimens. These fibres are very variable in diameter, usually from $\frac{1}{5000}$ to $\frac{1}{10000}$ of an inch in diameter.

"They bifurcate, or even divide into three; and the sum of the diameters of the branches considerably exceeds the diameter of the trunk. They anastomose freely with each other. They are prone to break under manipulation, and the broken extremities are abrupt and disposed to curl up: when many of these broken ends exists together in the same piece, they give it a very peculiar and characteristic appearance.

"In the human subject we find this tissue employed in the spine, as the *ligamenta subflava*, extended between the laminæ of the vertebræ; in the larynx forming the thyro-hyoid and crico-thyroid membranes, and the chordæ vocales; and the trachea, forming the longitudinal or elastic bands of that tube and its branches. The internal lateral ligament of the lower jaw, the stylo-hyoid ligament, and the transversalis fascia of the abdomen, are also, in a great measure, composed of it." It is evident that in all the situations here indicated only very controuled forces extend the elastic tissue.

"In *chemical constitution*, this tissue differs remarkably from the white fibrous tissue. It is unaffected by the weaker acids, or by boiling, and will resist putrefaction, and preserve its elasticity during a very long period. Very long boiling appears to extract from it a minute quantity of a substance allied to gelatine; but this is perhaps derived from the areolar tissue and vessels, which always penetrate sparingly among its fibres, and cannot be separated by dissection.

"We have hitherto spoken of the two forms of fibrous tissue as they occur in isolated masses; but their distribution through the body is far more extensive than this description would imply. In a diffused form, blended with one another in very varying proportions, and each one of them presenting a variety of modifications, they compose the *areolar tissue*."

"The areolar tissue" is very widely dispersed among the other tissues of the body, and of itself constitutes a principal portion of some organs. It serves the most important purposes in the *construction* of the body, by binding together, and yet allowing movement between, its elementary parts; and it contributes largely to the formation of membranes, conferring protection by their toughness, resistance, and elasticity.

"*Microscopic Characters.*—When a fragment of the areolar tissue from a favorable situation is examined, it presents an inextricable interlacement of tortuous and wavy threads intersecting one another in every possible direction. They are of two kinds. The first are chiefly in the form of bands of very unequal thickness, and inelastic. Numerous streaks are visible in them, not usually parallel with the border, though taking a general longitudinal direction. These streaks, like the bands themselves, have a wavy character, but they are rendered straight by being stretched. The streaks seem more the marks of a longitudinal creasing, than a true separation into threads; for it is impossible by any art to tear up the band into filaments of a determinate size, although it manifests a decided tendency to tear lengthwise.

"The larger of these bands are often as wide as $\frac{1}{500}$ of an inch; they branch or unite with others here and there. The smaller ones are often too minute to be visible, except with a good instrument. These are the *white fibrous element.*

"The others are long, single, elastic, branched filaments, with a dark decided border, and disposed to curl when not put on the stretch. These interlace with the others, but appear to have no continuity of substance with them. They are for the most part about the $\frac{1}{8000}$ of an inch in thickness; but we often see, in the same specimen, others, of much greater density. These form the *yellow fibrous element.*

"By the endless crossing and twining of these microscopic filaments, and of fasciculi of them, among one another, a web of amazing intricacy results, of which the interstices are most irregular in size and shape, and all necessarily communicate with one another."

"The areolar tissue is one of the most extensively diffused of all the elements of organization, and its chief purpose seems to be that of connecting together other tissues in such a way as to permit a greater or less freedom of motion between them. To do this, it is placed in their interstices, and is more or less *lax*, more or less abundant, according to the particular exigency of the part. It is by means of this tissue, as well as by the complexity of its own web, that almost every part of the vascular system is fixed in its position, and allowed to undergo the movements impressed upon it by the circulative powers. Even the capillaries supplying this system itself are for the most part brought to it, and enveloped, by this tissue."

"It is remarkable, however, that the central organ of the circulation, like the central organ of the nervous system, contains this tissue in very small proportion; one reason of which seems to be, that its fibres differ from the parallel fibres of other muscles, by twining among one another, and thus are enabled to dispense with an extraneous bond of connexion."

"Where great elasticity is required, the yellow element predominates; while the white fibrous element abounds in parts demanding tenacity and power of resistance. In all cases the openness of the network is proportioned to the extent of mobility required."

The retractor ligaments of the lion's claws, like the elastic coats of arteries, are set to counteract muscle at peculiar advantage. They yield momentarily, and operate efficiently as resistance declines. We cannot doubt that excessive tension converts the elastic tissues into the ligamentous, and this even in healthy bodies. Disuse brings fascia to reticular tissue. Free use and due nutrition may develop greatly the elastic, but beyond this condensation must follow.

From illustrations to be found in animals it would almost appear that—with altered blood and tensions—ligament may become elastic periodically—and the same applies even to our bodies.

Comparative Anatomy would furnish very various illustrations of elastic organized structure, and of its peculiar circumstances as to tension, but it is needless and hurtful to travel far from our proper provinces.

When we regard periosteum in many different parts of the system, including endosteum, the lining of bony cells and passages, and also the dura mater and spinal theca, we discover evidences of various specific grades or forms of tension. Vibration, the spreading insertion of muscles, the absence of tensions within, and the weak spongy tissue of some bones, offer considerable distinctions. The posterior half of the spinal theca throughout its length is the thicker by much, and is more elastic* than elsewhere. It struck me that it is also more subject to a guarded occasional stretching, just as the flavous ligament of the arches is, and the nuchal rope which helps to sustain the head of the horse. Now, considering that the other parts of the same theca are dense, thin, and ligamentous, I conceive the elasticity of the posterior half to be strikingly connected with its different relation to the centre of motion in the spine, with its freedom from adhesions, and its subjection to *specific* extensions.

The case of the Dura Mater may seem somewhat problematical. A dense tendinous sheet stretched between fixed points, and not without notable traits of habitual tension, well nourished, with fibres indicating very precisely the direction in which extensions act most energetically, and, though thin in health, certainly devoid of all appearances of atrophy or disuse;—what are the forces that may act upon it? The weight of the brain on the tentorium is all sustained by the great falx—may it not be that a tension equal to about one pound's weight—habitually operating through the day, (at the summit of the vascular column) with altered circumstances at night, serves to keep up the solidity of this protecting envelop? I would not estimate lowly the vascular distending force, though its operation varies. This is the only power by which I can explain the strong adhesion of the dura mater to the skull, and the first cause with respect to morbid forms of the adhesion. The solid cords of nerves subject to extensions without the skull, and the coatless feeble medullary cords within it, are in strong contrast.

The adhesions in the arachnoid are *close*, or short *films*; some are longer in the spinal canal.

Neurilema may be said to resemble ligament, in arising from bone and tendon, in having one extremity, in some way or other, subject to the indirect extension of muscles. The long and deflected course of nerves is however peculiar, and their measure of elasticity, though little, is distinct. The sheath of the optic nerve is in a certain sense only the ultimate insertion of two muscles of the globe. Vascular impulse in the orbit tends to project the ball. The great cause of a solid persistent sclerotica and cornea is, as I consider, the compression (or distension) caused by its muscles—nutrition being proportioned to resistance. The circumstances of the testes seem to be similar when they are exposed to pressures, and the albuginea, when in the abdomen of the adult, is thinner.

The fibrous tunics of the viscera† would appear feebler in proportion as they are little extended. The serous are prone to morbid thickenings from distension, attrition, bad nutrition, &c. If the strength and elasticity of the soft tissues of the air tubes are a criterion of the forces elongating them—which I scarcely doubt—I think we may adduce new and useful views of chest functions from them. The walls of the larynx seem to be gently stretched into form. Its egg-shell *bones* have a little to resist (like the coarse-celled sternum). The tracheal rings and the crescents in the bronchial bifurcations are very singular in form and office. They may become *earthy*, as rib-cartilages; not, I think, cancellated. So, if the vasa deferentia, ureters, gall-ducts, pancreatic, &c. indicate the forces obstructing their several orifices, but I would also anticipate exceptional conditions.

* The observation that the posterior half of the theca is elastic, is, as far as I know, original with Mr. J. Hilton, and it was in one of his dissections that I first saw strongly marked the following fact:—

The spindle-point at the lower end of the spinal cord sends an elastic cord to be attached down the sacral canal, and this seems to me to have just about the same kind of extension as the above.

† There seem to be grades of texture in the proper tunics of the lung, liver, spleen, thyroid, kidney, prostate, parotid, &c. varying compressions and distensions must both be taken into the account.

I return once more to the nerves. What firm threads and dense knots do the sympathetics present! but if extension may explain the hard tunic of each little cord, the tractions of many cords may account for the solid coats of the ganglia. The great semilunar ganglia have each at times (Mr. Swan) large unmeaning *rope's ends* attached to the supra-renal bodies; may not these likewise be the useful and physical result of traction on tissue, though atrophied nerve-cord originally. There are peculiar and distinct signs of atrophy and tensions internally and externally in the supra-renal bodies.

The elasticity of small aneurisms is I think greatest remotest from the heart. The tissue of valves compared with their vessels is remarkable. The valve is the more rarely and the more violently used—it looks like a compound of atrophy and hardness.—(*See* "Surfaces of Contact and Attrition on the Valves of the Heart," &c., *Guy's Hosp. Rep.* No. 10.) Various valves, arteries and veins *naturally* waste, without use*—the *efficient cause* of the persistence of others. The structure of vessels in different parts follows their use, while it indicates their need, as at the vertex and the foot. The casual and temporary changes of vessels have other causes.† The inferior cava being obstructed, all below will be distended, but the next most open course for the blood will be the most liable to occasional or momentary reliefs, and it is that which becomes enlarged to good purpose.

Comparing the elastic saphena vein with the dense cava—considering them as parts of the same system and even of the same cord, nay, in evolution almost identically one and the same things, can we see any more likely cause for the peculiar texture of each than the physical circumstances to which each is subject. The one valveless, liable to reflux and distensions sometimes violent, and always —with good nutrition—firm and equal to its wants; the other, extremely elastic, emptied by every motion, subject to some stretching similarly to the elastic theca of the spinal marrow, but perhaps subject to no violence either within or longitudinally.

I may here glance at the state of varices which seem yet little understood. The disorder is one of daily over distensions and imperfect nightly repairs—of a chronic or rather of a *variable* character. The greatest exertion with due repair makes both tubes and valves equal to their offices. The time is gone for cure, when the veins, being too wide, thick, hard and deformed, the valves also deteriorated and useless, and the skin defective as support, we set about getting rid of the channels altogether. The incipient, variable and perhaps most painful stages, when some balance between distension and nutrition (as to degree and quality) might avail, never seem to have been noticed.

The varied texture in different muscles results from their degree of use. It may not at first sight appear that the fasciæ owe their permanent solidity to habitual tension as ligament does, yet we may compare their tension to that of other tissues, as dura mater. One source of stretching (the margins being fixed) is found in lateral pressure, which thus tends to elongate the tissue and to determine the course of its dense threads. Aponeuroses, like neurilema, are subject to counter-extension by their more moveable bony connections through the agency of muscle. It is certain that fasciæ in general are only developed by muscular efforts, and that in their rise, seat, substance, and arrangement they are strictly

---

* The persistence of ductus arteriosus or cord gives the measure of the forces acting on it.

† I have formerly endeavoured to shew how it is that unusually easy circulation in a part empties and relaxes and so enlarges the artery of supply, and I have intimated the effect of healthy blood in such a case. The new and free course of the blood after tying a great artery was represented as a very simple problem. (London and Edin. Monthly, July 1842.)

governed by muscular extensions—they are equal to the positive insertions of tendons or of muscles.

Whilst ligament depends on strong tension, elasticity seems to result from a force of extension which is more gentle, gradual, and guarded, and more limited in its range of variation.

Ligament* may have the more complete temporary relaxation.

In conclusion it does not seem insignificant to remark, that while there is reason to infer that the blood takes up and deposits according to fixed rules of chemical attraction in dependance on the state of the circulating fluid, and on that of the organ or tissue nourished, we also discover manifest traces of other controlling or concurrent molecular forces. The state of repletion—compression of the fluids in the vessels, and the proportion of tension in particular tissues for the time being, manifestly affect the interchanges which the capillary blood undergoes. Changes of temperature depend on molecular changes, or *vice versâ*, produce such changes. Elasticity of tegument is regulated by vascular supply, belongs to and is essential to health, yet with external cold it may almost disappear.

That pressure on the blood regulates absorption and deposit generally is doubtless true, so also is it that extravascular tensions decide similar changes locally.

---

### Some Hints on the most Efficient Modes of Administering Medicines. By A Practitioner of Half a Century.

Many of the most important discoveries and improvements in medical science are rendered comparatively useless, in consequence of being unskilfully applied to actual practice. In no department of knowledge is this defect more conspicuous than in therapeutics. Man, (and I believe the same remark applies to all created beings), is born with a kind of instinctive antipathy to physick, which antipathy he retains from the cradle to the grave. Look at the ingenious spoons that have been invented to force physick down the throats of infants! Observe the mantel-pieces of sick chambers, and count how many phials are either uncorked, or only half emptied! How great a proportion of mankind hate the very name of physick! If the stomach is apt to turn at the thought of medicine, when we are in health, how much less capable is it to bear nauseous drugs in the various forms of disease, nine tenths of which affect the stomach sympathetically with squeamishness, nausea, and aversion to food as well as physick? The evil consequences of nauseous forms of medicine being used in sickness, are great beyond all calculation or belief. One result is, that medicine is not taken in sufficient quantity—sufficiently often—or for a proper length of time.

What practitioner will fail to recognise the following picture of almost daily occurrence. A medical man is in anxious attendance on a patient—say a lady after confinement, and threatened with some grave malady—peritonitis, for instance. He prescribes what he conceives to be active and efficient remedies for the night, and gives strict injunctions to the nurse. In the morning, when he calls, he meets the nurse on the stairs. Have you given your mistress the medicines punctually? Most punctually, Sir. Well, what has been the effect? "Brought everything up again Sir." What, all? "Every drop, Sir—and I

* It seems to me extremely probable that every valve and every ligament is developed in its proper place by the very physical force which subsequently demands such aid. The sites of venous valves in this respect are very remarkable. The valves and the tunics of lymphatics are all but equal, and so are their tensions; the currents being easy and rapid.

thought she would have brought her very heart up with it." After such intelligence, the feelings of the doctor, on entering the chamber, are not particularly enviable. Now all this is more frequently owing to the form than the substance of the medicine exhibited.

In chronic diseases, where the remedial process is necessarily chronic also, we are daily baffled by the repugnance—nay, the resistance of the patient to a protracted course of physic. Yet it might very generally be so contrived, that the patient would desire rather than loathe his medicines!

I am aware that in some acute diseases, the state of nausea itself is desirable, and salutary. But it is not the mere nausea or sickness which lessens the velocity of the circulation, opens the secretory vessels, and checks inflammation. These remedial processes depend much upon the *quantity* of medicine—say antimony—which the patient can bear in order to induce them. Thus double or triple the quantity of tartrate of antimony will be borne, before sickness is induced, if given in an effervescing draught, as compared with the same medicine given in plain water. And the remedial effects will be in proportion. This is a truth that should ever be held in mind, and the principle was well understood by Rasori, Thomasini, and others. The contra-stimulant effects of antimony are trifling during the nausea and sickness at the beginning:—it is when the *tolerance* is acquired that the inflammation or high fever is controlled.

But there is a large class of diseases in which the stomach is morbidly irritable, and where nauseating medicines are positively injurious. Putting aside the multitudinous forms of dyspepsia, we have affections of the uterus, the kidney, the liver, the pancreas, &c., where the stomach is prone to disordered function, and where it is of the greatest consequence to exhibit medicines in forms that will tranquillize rather than nauseate the stomach. Diseases and disorders of the kidney are now acknowledged to be much more frequent than they were formerly suspected to be—and these are very generally attended with gastric irritability. In these it is of great importance not to ruffle the stomach by medicines. In affections of the brain, now so exceedingly common in consequence of the advanced stage of civilization, and the operation of various perturbing moral causes, the stomach is often the organ most conspicuously deranged—and we are not seldom foiled in the exhibition and perseverance of proper remedies, from the sympathetic disorder of stomach.

Nine-tenths of the cures that are said to be performed by homœopathy, result from the spare diet and the nullity, as it were, of medicine employed. Of all the medicines that are prescribed by the physician, the class of salines are the most generally beneficial, as opening the secretory organs, as the skin, the liver, the kidneys, &c., besides improving the state of the blood, and restraining febrile action in the constitution. These, when exhibited in an effervescing state, are far more palatable, as well as more efficacious, than when given in a plain form.

Tonics, on which the routine practitioner so much relies, and which he exhibits with no sparing hand, are more frequently injurious than beneficial. They give a feeling of tone for a time; but they lock up the secretions, increase too much the appetite, and lay the foundation for future states of plethora, congestion, or indigestion.

Now saline effervescents may be made the vehicle for many of the most powerful tonics, and indeed the most potent medicines which we possess. The citrate of iron, colchicum, antimony, arsenic, quinine, iodine, &c. &c., may all be exhibited in a form that increases their remedial efficiency, and lessens their tendency to nauseate the stomach.

Senex.

